# Evaluating the predictive accuracy of curated biological pathways in a public knowledgebase

**DOI:** 10.1093/database/baac009

**Published:** 2022-03-06

**Authors:** Adam J Wright, Marija Orlic-Milacic, Karen Rothfels, Joel Weiser, Quang M Trinh, Bijay Jassal, Robin A Haw, Lincoln D Stein

**Affiliations:** Adaptive Oncology Program, Ontario Institute for Cancer Research, 661 University Avenue Suite 500, Toronto, ON M5G 0A3, Canada; Adaptive Oncology Program, Ontario Institute for Cancer Research, 661 University Avenue Suite 500, Toronto, ON M5G 0A3, Canada; Adaptive Oncology Program, Ontario Institute for Cancer Research, 661 University Avenue Suite 500, Toronto, ON M5G 0A3, Canada; Adaptive Oncology Program, Ontario Institute for Cancer Research, 661 University Avenue Suite 500, Toronto, ON M5G 0A3, Canada; Adaptive Oncology Program, Ontario Institute for Cancer Research, 661 University Avenue Suite 500, Toronto, ON M5G 0A3, Canada; Adaptive Oncology Program, Ontario Institute for Cancer Research, 661 University Avenue Suite 500, Toronto, ON M5G 0A3, Canada; Adaptive Oncology Program, Ontario Institute for Cancer Research, 661 University Avenue Suite 500, Toronto, ON M5G 0A3, Canada; Adaptive Oncology Program, Ontario Institute for Cancer Research, 661 University Avenue Suite 500, Toronto, ON M5G 0A3, Canada; Department of Molecular Genetics, University of Toronto, Room 4396, Medical Sciences Building, 1 King’s College Circle, Toronto, ON M5S 1A1, Canada

## Abstract

**Abstract:**

Reactome is a database of human biological pathways manually curated from the primary literature and peer-reviewed by experts. To evaluate the utility of Reactome pathways for predicting functional consequences of genetic perturbations, we compared predictions of perturbation effects based on Reactome pathways against published empirical observations. Ten cancer-relevant Reactome pathways, representing diverse biological processes such as signal transduction, cell division, DNA repair and transcriptional regulation, were selected for testing. For each pathway, root input nodes and key pathway outputs were defined. We then used pathway-diagram-derived logic graphs to predict, either by inspection by biocurators or using a novel algorithm MP-BioPath, the effects of bidirectional perturbations (upregulation/activation or downregulation/inhibition) of single root inputs on the status of key outputs. These predictions were then compared to published empirical tests. In total, 4968 test cases were analyzed across 10 pathways, of which 847 were supported by published empirical findings. Out of the 847 test cases, curators’ predictions agreed with the experimental evidence in 670 and disagreed in 177 cases, resulting in ∼81% overall accuracy. MP-BioPath predictions agreed with experimental evidence for 625 and disagreed for 222 test cases, resulting in ∼75% overall accuracy. The expected accuracy of random guessing was 33%. Per-pathway accuracy did not correlate with the number of pathway edges nor the number of pathway nodes but varied across pathways, ranging from 56% (curator)/44% (MP-BioPath) for ‘Mitotic G1 phase and G1/S transition’ to 100% (curator)/94% (MP-BioPath) for ‘RAF/MAP kinase cascade’. This study highlights the potential of pathway databases such as Reactome in modeling genetic perturbations, promoting standardization of experimental pathway activity readout and supporting hypothesis-driven research by revealing relationships between pathway inputs and outputs that have not yet been directly experimentally tested.

**Database URL:**

www.reactome.org

## Introduction

Reactome is a manually curated and peer-reviewed open-source database of human biological pathways (1). The pathway annotation process follows a deliberative procedure of human curation, internal review and external peer review steps to create accurate, unambiguous and auditable assertions. Reactome curators and editors are PhD-level biologists and biochemists with substantial wet lab experience. Therefore, they are highly skilled in the critical assessment of published experimental evidence and synthesis of scattered, experimentally-derived knowledge into coherent biological pathways. The process of manual curation is enhanced by the participation of scientists who volunteer to add additional annotations to the pathways based on their domain of expertise. Reactome’s internal review steps involve independent checking of annotations for comprehensiveness and consistency by curators and editors who were not involved in the annotation process. Finally, Reactome’s peer review process involves checking the pathway content for completeness, accuracy and the absence of obvious bias by scientific domain experts (2, 3). Reactome is updated and released quarterly. Long-term curation goals and yearly curation priorities aim to set a balance between adding new and updating existing annotations (4).

In line with other manually curated biological databases (5), the accuracy of Reactome’s annotations at the reaction level is expected to be high due to manual expert curation, peer review, and several manual and automated quality assurance steps. However, despite heavy usage of Reactome by the international research community for gene set enrichment analysis and pathway/network analysis, the utility of Reactome for predicting the functional effects of perturbing pathway components has never been formally evaluated. Here we present a study that compares Reactome pathway diagram-based predictions of the effects of bidirectional perturbations (upregulation/activation or downregulation/inhibition) of pathway inputs on the activation state of key pathway outputs against published empirical results using a novel algorithm, MP-BioPath. For comparison, we also look at the accuracy of curators’ perturbation effect predictions using the same reference pathway diagrams applied to MP-BioPath.

## Materials and Methods

### Reactome pathway selection

The central unit of annotation in Reactome is a *reaction*, a biological event defined by a unique combination of inputs, outputs, catalysts and regulators. A reaction in Reactome is always supported by direct experimental evidence in a human research model or inferred from an orthologous reaction that is supported by direct experimental evidence in a model organism (6). Reactions connected by shared inputs and outputs are grouped into causal chains to form biological pathways (2). These pathways are stored in a machine-readable data structure and converted in an automated fashion into human-readable pathway diagrams for display on the Reactome website and associated tools.

To evaluate the predictive power of Reactome pathways, we selected 10 cancer-relevant Reactome pathways from version 66 (V66, June 29, 2018), representing an array of biological processes such as signal transduction, cell division, DNA repair and transcriptional regulation (Table 1). These 10 pathways were selected because of their enrichment for genes frequently mutated in large public breast, colorectal and pancreatic genomic datasets (7), their curation status (first published or revised within 20 Reactome release cycles before V66) and the diversity of pathway structures, participants and outcomes. Seventy-three additional pathways, containing over 3500 unique genes, that were similarly enriched for cancer driver genes were selected to optimize the MP-BioPath tool, as described below.

**Table 1.** Ten cancer-relevant Reactome pathways from version 66 (V66) selected for evaluation of the predictive accuracy of Reactome pathways

Reactome pathway identifier	Reactome pathway name	Selected root inputs	Selected key outputs	Test cases	Test cases with available published evidence	Total number of nodes	Total number of edges
68 875	Mitotic Prophase	12	11	264	26	262	273
69 242	S Phase	11	9	198	25	409	437
69 620	Cell Cycle Checkpoints	7	14	196	55	511	552
195 721	Signaling by WNT	7	37	518	49	817	953
453 279	Mitotic G1 phase and G1/S transition	17	26	884	89	431	556
1 227 986	Signaling by ERBB2	10	9	180	49	212	246
1 257 604	PIP3 activates AKT signaling	16	14	448	200	781	1066
3 700 989	Transcriptional Regulation by TP53	18	51	1836	257	1106	1276
5 673 001	RAF MAP kinase cascade	7	6	84	49	641	834
5 693 567	HDR through Homologous Recombination (HRR) or Single Strand Annealing (SSA)	18	10	360	48	279	310

### Converting reactome pathways to logical networks

The 10 selected Reactome pathways were converted to logic graph formats (from now on called Logical Networks) to facilitate semi-automated pathway analysis (Supplementary Figure S1 and Supplementary Table S2). The script used to convert pathways stored in the Reactome database into Logical Networks is available in GitHub (https://github.com/reactome/Release/blob/master/scripts/reaction_logic_table.pl). A detailed description of the conversion process is available in the Supplementary Methods.

Logical Networks consist of input (I) and output (O) node pairs and their connecting directed edges (described in Supplementary Methods). Root inputs (RI) in Logical Networks are input nodes (I) that never became outputs (O) in a pathway (RI = I—O), while terminal outputs (TO) are output nodes that never came to be inputs (TO = O—I). Curators used Cytoscape (8) to visualize generated Logical Networks.

### Selection of root input nodes and key pathway outputs

We selected RI nodes to use for perturbation predictions by intersecting the pathway gene set with the COSMIC database Cancer Gene Census (CGC) list (9) to find well-studied cancer-related genes. On several occasions, we selected as RIs relevant genes that were not part of the CGC (Supplementary Table S1). If several CGC genes belonging to one protein family were annotated as functionally interchangeable in a Reactome pathway, the best experimentally characterized gene was selected as the RI. Exceptions were made in the case of the well-studied genes AKT2 and MDM4, which were included alongside AKT1 and MDM2, respectively, and in the case of KRAS, NRAS and HRAS, and BRAF and RAF1. The category of molecules that RIs belonged to depended on the pathway—they could be either proteins, mRNAs or genes or some combination.

**Figure 1.**
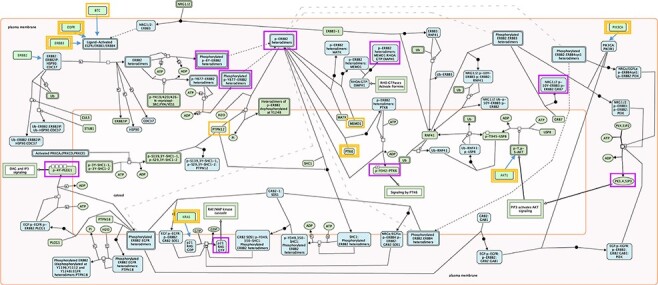
Root inputs and key outputs selected for Reactome pathway ‘Signaling by ERBB2’. Root inputs are circled orange, with double border if they are in Cancer Gene Census list. Key outputs are circled purple. If root inputs are not directly shown in the diagram they have been redrawn and connected with the root complex/set.

The selection of key outputs for each pathway was accomplished by finding all TO nodes indicative of pathway activation. TO nodes involved in negative feedback loops were excluded. Occasionally, nonterminal output nodes commonly used in biological assays to determine pathway activity were included in the testing. Key outputs could be individual proteins, mRNAs, small molecules, protein complexes, polymers, sets or, rarely, reaction nodes, when a TO was a part of a feedback loop. An example of RIs and key outputs is shown in Fig. 1. All selected RIs and key outputs are listed in Supplementary Table S1.

### Literature search

A manual search of the PubMed database was conducted for each test case, using RI name, key output name and perturbation type as keywords. Gene name synonyms were included in the search. Search results were manually filtered based on the abstract content, and full text and figures of selected original research articles were inspected for relevant experimental findings and author statements. Various experimental designs and readouts were taken into account, as listed in Supplementary Table S1. In total, 531 papers (one paper was retracted after this study was completed, as indicated in the table) contained citable experimental evidence relevant for 847 test cases (the evidence from the retracted paper was excluded) (Supplementary Table S1). Of these, 158 papers were previously cited by the Reactome database in the context of the 10 selected pathways (Supplementary Table S1). Reactome-cited papers were not excluded from this study since these papers frequently contained the best available mechanistic evidence, were approved as relevant by external experts during Reactome peer review, and sometimes represented the only published experimental evidence of perturbation effects available.

### Curator-based prediction of perturbation effects and inter-curator agreement

Inter-curator prediction agreement was determined between three curators on 100 randomly selected test cases by calculating percent agreement and the Fleiss Kappa statistic. Random selection of 100 test cases for inter-curator agreement determination was completed using Excel’s RAND function on 847 test cases with available literature evidence. Curators were trained on a pathway that was not used in the study to navigate Logical Networks in Cytoscape, apply the PathLinker tool to identify paths between RI and key outputs in Reactome Logical Networks, and predict effects of root input perturbations on key outputs (upregulation, downregulation and no change) as depicted in Supplementary Figure S2. Curators were blinded to each other’s predictions. Percent agreement and Fleiss’ Kappa were calculated manually in Excel and using ReCal, an online tool (10, 11).

Each test case consisted of a unique combination of an RI, RI perturbation (upregulation or downregulation), a key output and the predicted change in the activity of the key output node (upregulated activity, downregulated activity or no change) based on the Logical Network relationships. To predict the effects of a perturbation of the RI on the key output state, three Reactome curators applied the following rules (1): If an RI was connected with a key output through the edges of exclusively positive polarity or an even number of edges of negative polarity, the RI perturbation was predicted to result in the key output perturbation of the same directionality (Supplementary Figure S2A) (2); If a path from an RI to a key output contained an odd number of edges of negative polarity, the RI perturbation was predicted to result in the key output perturbation in the opposite direction (Supplementary Figure S2B) (3); If there was no path from an RI to a key output, or if an RI was connected to a key output through two paths of opposing polarity, the RI perturbation was predicted to cause no perturbation in the key output (Supplementary Figure S2C) (4). When an RI and a key output were connected through more than two paths, the polarity of each individual path was determined and the polarity of the majority of connecting paths was assigned as the combined polarity of the RI-key output relationship (not shown). The PathLinker Cytoscape app (12) was used to facilitate identification of all paths between RI and key outputs in Logic Networks.

### Predicting effects of root input perturbations computationally

In order to computationally simulate the impact of perturbations to RIs we developed a new computational algorithm, MP-BioPath (https://github.com/OICR/mp-biopath), to automate predicting the impact of RI perturbations on key outputs in Reactome pathways. This algorithm uses the Logical Networks to develop a non-linear mathematical model for each of the pathways. A high-level description of the MP-BioPath software can be found in the Supplementary Methods.

MP-BioPath’s mathematical model uses continuous values instead of discrete states, which is applicable to a diverse set of biological use cases. Values for each node can range between 0.01 and 100, where 1 represents an unperturbed (normal) activity of the node, 0.01 represents a 100 times decreased activity compared to normal and 100 represents 100 times increase of the normal activity amount.

Input nodes are related to output nodes via non-linear equations that take into account the logic and polarity of connecting edges. When inputs are connected to the output with positive polarity edges of AND logic, input node values are multiplied to determine the output node value. When an input is connected to the output with a negative polarity edge, a reciprocal value of the input is used in the multiplication equation. When inputs are connected to the output with edges of OR logic, the value of the output node is calculated as an arithmetic average of the values of input nodes. In this way, the value of each input node is considered when computing the value of the output node.

**Figure 2.**
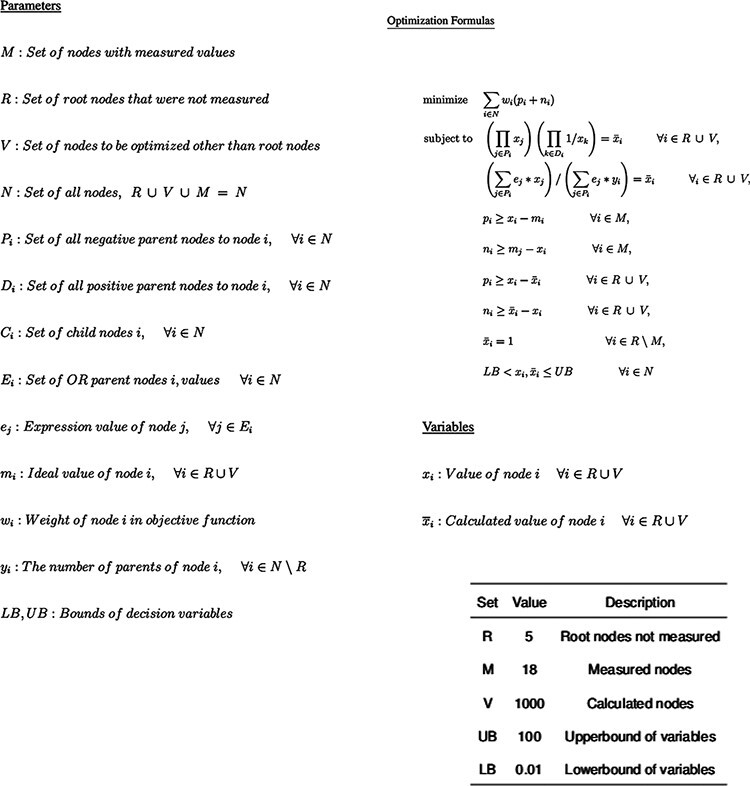
Optimization Model for inferring perturbation impact on pathway nodes. The top is the parameters available in the model, followed by the non-linear model itself and then by the fixed parameter values.

The resulting system of equations is aggregated into a single optimization model for each pathway in order to calculate the magnitude and direction of the change of all nodes in the pathway that result from fixing the values for the known input perturbations. As it is possible for circumstances to arise where there is no solution to the system of equations based on the known inputs, flexibility is built into the model by having two values for each node: $\bar x$ and $x$. In general, $\bar x$ is used as the input value to the equations and $x$ is used as the output value. The optimization model minimizes the difference between these two values. Although minimizing the square distance would spread the remaining disagreement across all nodes involved, the algorithm does not do this in the interest of reducing run time (Figure 2).

MP-BioPath emits continuous values for the activity level of key outputs. In order to compare these values to literature-based perturbation experiments we needed to discretize these values to upregulation, downregulation, and no change. To establish appropriate cutoffs, we evaluated a range of cutoff values against curator-generated predictions across 18 539 test cases from 73 Reactome pathways (Supplementary Table S4).

**Figure 3.**
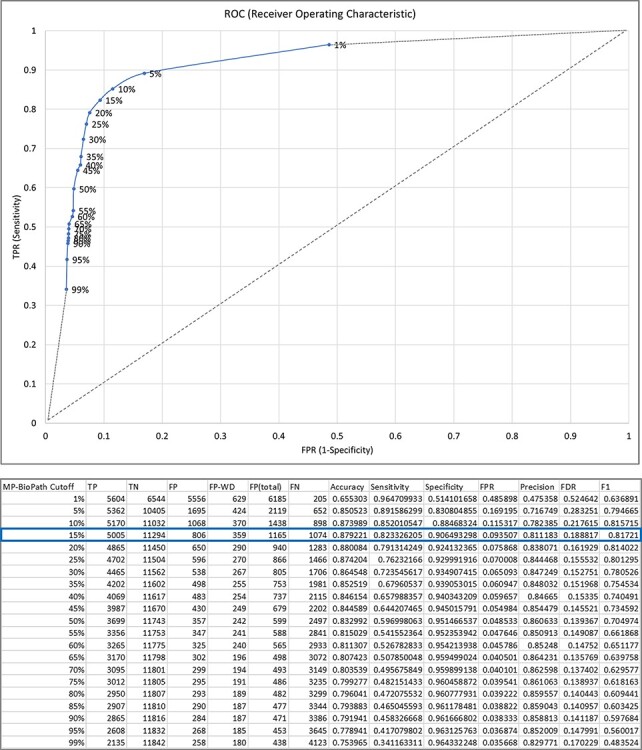
The relationship between MP-BioPath sensitivity (true positive rate, TPR) and false positive rate (FPR) at different cutoffs.

Treating the curator predictions as the ground truth, we evaluated cutoffs between 1% and 99% in 5% increments in order to construct an ROC curve and determine the accuracy, sensitivity, specificity, precision, false discovery rate (FDR) and F1 score at each cutoff (Figure 3). The F1 value was maximal for the cutoff of 15%. Therefore, upregulation/downregulation of 15% or more was used as the cutoff when converting MP-BioPath-computed change levels to discrete values for 847 test cases with published experimental evidence.

### Comparison of curator and computational predictions with published results

In total, 4968 test cases were analyzed over 10 pathways. Results of test cases for which experimental evidence was available were recorded and organized into a confusion matrix. Concordant test cases were scored either as true positives (TP) or true negatives (TN). A TP value was assigned when both Logical Networks and experimental evidence supported upregulation or downregulation of a key output as a consequence of an input perturbation. A TN value was assigned when the Logical Networks and experimental evidence supported no effect of an input perturbation on a key output. Discordant test cases were scored as either false negatives (FN) or false positives (FP). An FN value was assigned when, based on the Logical Networks, an input perturbation was predicted to not affect an output, but experimental evidence demonstrated that the output was affected. An FP value was assigned when, based on the Logical Networks, an input perturbation was predicted to affect the output, but experimental evidence showed that the output was not affected.

As perturbations were bidirectional, a special case of FP was a false positive-wrong direction (FP-WD). An FP-WD value was assigned when, from the Logical Networks, an input perturbation was predicted to affect the output in the opposite direction from the experimental evidence (e.g. the pathway Logical Network-based prediction was that the output would be downregulated, while experimental evidence demonstrated upregulation of the output).

The resulting confusion matrix was used to calculate the average sensitivity and accuracy of Reactome pathway annotations. The comparison of accuracy, sensitivity, and specificity between curator-based vs. MP-BioPath based predictions on 847 test cases with published experimental results was done by running the Mann–Whitney test using the online tool Social Science Statistics (13).

## Results

### Characteristics of selected pathways and test cases

The 10 selected pathways, representing more than 1000 unique genes, ranged in size from 212 nodes and 246 edges (Signaling by ERBB2) to 1106 nodes and 1276 edges (Transcriptional Regulation by TP53), while the number of test cases per pathway ranged from 84 to 1836 (Table 1). Some nodes and edges appear in more than one pathway (Supplementary Table S2) for a total of 4136 unique nodes, 5308 unique edges and 4968 test cases spanning 10 pathways. The number of edges between RIs and key outputs (path length) ranged from 1 to 46, with the majority of test cases falling into the distance range of 4–17 edges (Supplementary Figure S3). Published experimental evidence was available for 847 of these test cases. Long paths (>20 edges) between RIs and key outputs were significantly more prevalent among test cases for which published experimental evidence was not available (Supplementary Figure S4). The availability of experimental evidence for test cases in different pathways varied, ranging from 10 to 60%. This range is explained in part by the type of key outputs used as a readout of pathway activity and by the interconnectedness of the pathway with other biological processes. For example, key pathway outputs representing gene expression or protein phosphorylation are more amenable to experimental testing than the formation of large protein complexes or supramolecular structures, while pathways such as PI3K/AKT signaling and RAS/RAF/MAP kinase signaling, which lie downstream of many growth factor signaling pathways, are subject to more extensive experimental exploration as they are used as readouts for the activity of upstream pathways.

Of the 4968 test cases, one-half of perturbations (2484) represented upregulation and the remainder represented downregulation. Among the 847 test cases supported by published experimental evidence, 54% of RI perturbations represented downregulation, while 46% represented upregulation. In contrast, RI upregulation was slightly more frequent than downregulation among 4121 test cases with no supporting experimental evidence (51% and 49%, respectively). This is consistent with the observation that experimental studies preferentially use a loss-of-function approach to determine the roles of genes and their protein products, as previously reviewed (14).

With respect to the status of key pathway outputs in 4968 test cases, perturbations of RIs were predicted to result in the upregulation of key pathway outputs in 1332 test cases (27%), downregulation in 1334 cases (27%), and to have no effect in 2302 cases (46%). No effect of RI perturbation on the key output status was expected when there was no path between an RI and a key output in Logical Networks (90% of cases) or when a negative feedback loop existed between an RI and a key output (10% of curator-predicted no-effect cases). While a plurality (46%) of the total test cases were predicted to have no effect on key outputs, just 161 (19%) of the 847 test cases with experimental evidence were ones predicted to have no effect, presumably reflecting the well-documented bias towards positive results in the published literature (15, 16).

### Inter-curator agreement

To measure inter-curator agreement on predicted perturbation effects, we randomly selected 100 test cases covering all 10 pathways as listed in Supplementary Table S3. Complete agreement was achieved on 81/100 predictions and there were no cases in which all three curators disagreed (Supplementary Table S3). The average pairwise percent agreement was 87.3%, with the Fleiss’ Kappa of 0.8 and the average pairwise Cohen’s Kappa of 0.801 (Table 2). The Kappa value of 0.8 is in the range of substantial agreement (0.61–0.8) and is approaching the range of almost perfect agreement (0.81–1.00). When comparing three curator pairs, the highest agreement (97%, Cohen’s Kappa of 0.952) was achieved between the two curators who had a more extensive experience with the Logical Networks and the pathway content, as they were involved in the selection of key pathway outputs and in researching literature for relevant experimental evidence for the 4968 test cases.

**Table 2.** Summary of inter-curator agreement results

Number of testers	Number of cases	Number of decisions
3	100	300
Percent agreement		
Testers 1&3 pairwise agreement	Testers 1&2 pairwise agreement	Testers 2&3 pairwise agreement
81%	84%	97%
Average pairwise percent agreement		87.333%
Fleiss’ Kappa		
Observed agreement	Expected agreement	Fleiss’ Kappa
0.873	0.367	0.8
Cohen’s Kappa (CK)		
Testers 1&3 pairwise CK	Testers 1&2 pairwise CK	Testers 2&3 pairwise CK
0.703	0.749	0.952
Average pairwise CK		0.801

As the inter-curator agreement was substantial, we divided the workload of predicting the outcome of RI perturbations across the remaining 747 test cases among curators. We similarly divided the workload for prediction of effects for the 18 539 test cases across 73 pathways used to evaluate MP-BioPath cutoffs as described in the next section.

### Concordance of Reactome pathway-based perturbation effect predictions with published experimental evidence

Of the 847 test cases with experimental evidence, curator predictions based on Logical Networks agreed with published evidence in 670 cases (81% concordance), while MP-BioPath predictions were concordant with the published evidence in 625 test cases (75% concordance) (Table 3; Supplementary Table S1). The average sensitivity of curator-generated and MP-BioPath-generated predictions was 85% and 78%, the average specificity 70% and 62% and the average precision 92% and 88%, respectively. However, these values varied considerably across pathways. The prediction accuracy for the 10 selected pathways ranged from 56% to 100% for curators and from 44% to 94% for MP-BioPath. The most accurate predictions were for the 49 test cases with experimental evidence derived from the ‘RAF/MAP kinase cascade’, which had no false positives or negatives among curator-based predictions, and had three false positives and no false negatives among MP-BioPath-based predictions, achieving 100% and 94% accuracy respectively. The worst performance was observed in ‘Mitotic G1-G1/S Phases’, which had an accuracy of just 56% and 44% for curator-based and MP-BioPath-based predictions, respectively. Results for each pathway are summarized in Table 3.

**Table 3.** Concordance of Reactome pathway-based perturbation effect predictions with experimental evidence

	Reactome pathway name	TP		TN		FP total (FP-WD)		FN		Accuracy (%)		Sensitivity (%)		Specificity (%)		Precision (%)	
Reactome pathway identifier		C	MPB	C	MPB	C	MPB	C	MPB	C	MPB	C	MPB	C	MPB	C	MPB
68 875	Mitotic Prophase	23	21	1	0	1 (1)	4 (3)	1	1	92	81	96	95	50	NA	96	84
69 242	S Phase	17	14	2	2	0 (0)	1 (1)	6	8	76	64	74	64	100	67	100	93
69 620	Cell Cycle Checkpoints	42	42	5	5	2 (2)	2 (2)	6	6	85	85	88	88	71	71	95	95
195 721	Signaling by WNT	36	29	2	1	0 (0)	2 (1)	11	17	78	61	77	63	100	33	100	94
453 279	Mitotic G1-G1/S phases	40	16	10	23	29(6)	13 (3)	10	37	56	44	80	30	26	64	58	55
1 227 986	Signaling by ERBB2	36	33	4	4	0 (0)	0 (0)	9	12	82	76	80	73	100	100	100	100
1 257 604	PIP3 activates AKT signaling	185	173	0	0	14 (7)	17 (10)	1	10	93	87	99	95	NA	NA	93	91
3 700 989	Transcriptional Regulation by TP53	156	157	25	16	16(12)	26 (13)	60	58	70	67	72	73	61	38	91	86
5 673 001	RAF/MAP kinase cascade	48	45	1	1	0 (0)	3 (3)	0	0	100	94	100	100	100	NA	100	94
5 693 567	HDR through Homologous Recombination (HRR) or Single Strand Annealing (SSA)	36	43	1	0	4 (2)	5 (2)	7	0	77	90	84	100	20	NA	90	90
Total		619	573	51	52	66 (30)	73 (38)	111	149								
Average ± SD										81 ± 13	75 ± 16	85 ± 10	78 ± 22	70 ± 33	62 ± 24	92 ± 13	88 ± 12

TP = true positive; TN = true negative; FP = false positive; FP-WD = false positive—wrong direction; FN = false negative; C = curator-based predictions; MPB = MP-BioPath-based predictions.

The predictive accuracy of either curators or MP-BioPath was unaffected by the size of the pathway, measured either by its total number of edges or nodes (Supplementary Table S8), nor by the number of citations per pathway or node.

We noted a modest positive correlation trend between the prediction accuracy and the number of citations per reaction-like event, but this effect did not achieve statistical significance (Supplementary Figure S6). No significant correlation was found between prediction accuracy and the proportion of Reactome pathway citations that were also used as published evidence for effect of RI perturbations on the status of key outputs (Supplementary Figure S6A-D).

When considering the types of reaction-like events present in different pathways, e.g. association, dissociation, conversion, posttranslational modification (PTM) and gene expression regulation (GER) (17), for the 10 pathways of interest (Supplementary Table S7), we examined whether the overall proportion and ratio of PTM and GER events affected prediction accuracy as these event types frequently introduce a more complex pathway topology, such as feedback loops and a multitude of paths between a pathway input and a pathway output. We found that the accuracies of curator-based and MP-BioPath-based predictions were positively correlated with the proportion of PTM events, reaching significance for curator-based predictions. In contrast, the accuracies of curator-based and MP-BioPath-based predictions were significantly negatively correlated with the proportion of GER events, and even more significantly with the ratio of GER to PTM events in a pathway (Supplementary Figure S6E-G). This implies that the annotations of GER events relative to PTM events may be less complete and/or less supported by the Reactome data model, as discussed below.

In addition to analyzing the overall accuracy of curator-based and MP-BioPath-based predictions, we took a deeper dive into the ability of curators and MP-BioPath to predict the response to perturbations. In this analysis, we stratified test cases by their effect type (up, down and no change) as shown in Supplementary Table S5. Curators were 88%, 90% and 84% accurate in predicting upregulation, downregulation and no change respectively. The respective accuracies of MP-BioPath were 84%, 86% and 79%. There was no significant difference in the accuracy, sensitivity and specificity between curators and MP-BioPath when predicting either upregulation, downregulation or no change (Supplementary Table S6; Mann–Whitney test).

### Analysis of discordant test cases

**Figure 4.**
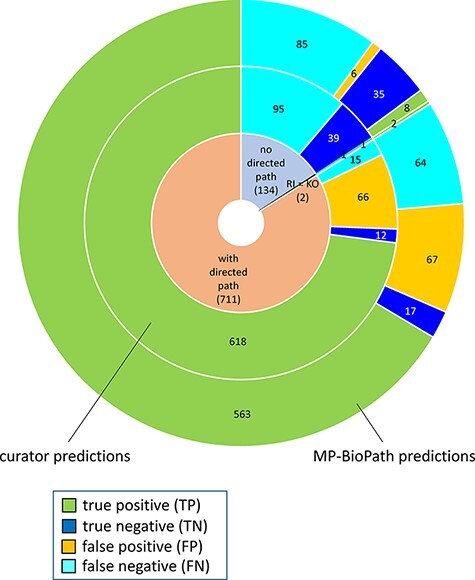
Distribution of true positives, true negatives, false positives and false negatives with respect to existence of directed path between a root input and a key output in Reactome pathway diagrams RI = root input; KO = key output.

**Figure 5.**
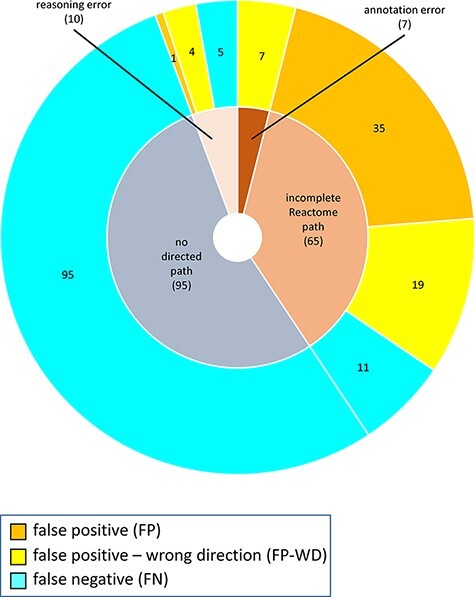
Reasons for discordance between curator-based predictions and published evidence.

The distribution of confusion matrix categories with respect to the existence of a directed path between RI and key outputs in Reactome pathway diagrams is shown in Fig. 4. For curator-based predictions, TP and FP values were exclusively assigned to test cases where a path could be established between a root input and a key output, while TN and FN values were generally assigned to test cases where no path could be established between a root input and a key output. This implies that all FP cases and a small number of FN cases are caused by incomplete or inaccurate annotations—for example, missing participants and regulators in an existing path, or wrongly assigned edge polarity in an existing path. FN cases, however, mainly result from missing annotations, when the knowledgebase does not include an established path between participating nodes. A more detailed analysis of erroneous curator predictions (Figure 5) shows that FP-WD cases were attributable to incomplete Reactome paths (19/30, 63%), errors in existing Reactome annotations (7/30, 23%) and curators’ reasoning errors during prediction (4/30, 13%). FP predictions in the strict sense were due to incomplete Reactome paths (35/36, 97%), and reasoning errors (1/36; 3%). FN predictions could be explained by non-existing directed paths between RI and key outputs (95/111; 85%), incomplete existing paths (11/111; 10%), and reasoning errors during prediction making (5/100; 5%). Only 5% of discordant cases (7 tests total) could be attributed to database annotation errors.

**Figure 6.**
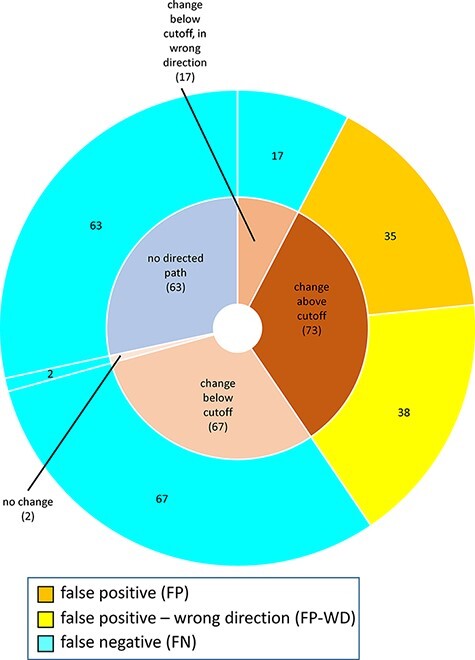
Reasons for discordance between MP-BioPath-based predictions and published evidence.

For MP-BioPath-based predictions, all FP and FP-WD cases were due to calculated changes above the activity value cutoff. The plurality of FN cases were due to calculated changes in the right direction, but below the cutoff (67/149; 45%), followed by test cases with no directed path (63/149; 42%), calculated changes below the cutoff but in the wrong direction (17/149; 11%), and, lastly test cases with no change (2/149; 1%; Figure 6).

The overlap between curator-derived and MP-BioPath-derived predictions shows a high degree of correlation among TP, TN and FN classes, consistent with most FN errors resulting from incomplete pathway coverage in the knowledgebase, and lower correlation for FP and FP-WD cases consistent with different sources of error for these cases (Supplementary Figure S7).

The distribution of path lengths did not differ significantly between the concordant and discordant test cases for either curator-based or MP-BioPath-based predictions (Supplementary Figure S4). When comparing path lengths between different confusion matrix categories, a significantly different distribution was detected between curator-derived TP and TN, TP and FN, FP and TN, and FP and FN cases. For MP-BioPath-derived confusion matrix categories, a significantly different distribution of path lengths was found between TP and FN, FP and FN, and FN and TN cases. The median and average path lengths for all confusion matrix categories, however, were similar, irrespective of whether they were curator- or MP-BioPath-derived (Supplementary Figure S5). It should be noted that the number of test cases in the TP category surpassed the number of test cases in other categories on the order of 10–30-fold.

## Discussion

Manual curation of the biological literature has long been considered to be the gold standard for pathway knowledgebases, and these curated knowledgebases are popular for data interpretation, analysis and hypothesis generation (18). Multiple studies have attested to the accuracy of manual curation (5, 19–21), and pathway analysis is widely used to interpret the results of ‘omic-scale studies.

While it is assumed that the knowledge gathered in curated pathway databases has utility for predicting the effect of naturally occurring and experimental perturbations, it is somewhat surprising that this assumption has never been empirically tested. In this study, we directly assessed the ability of Reactome, a large open access knowledgebase of curated human biological pathways, to predict the empirically-ascertained consequences of biological perturbations, by comparing Reactome pathway-based conclusions against published literature observations. Using a semi-automated process, we generated pathway Logical Networks for 10 cancer-related pathways covering ∼1200 unique genes and, from these graphs, we made nearly 5000 predictions of the downstream consequences of either increasing or decreasing the activities of pathway RI. The predictions were generated both manually, by curators, and computationally, using the novel pathway modeling tool MP-BioPath. We then mined the published literature to identify empirical studies that directly examined the effects of 847 of these perturbations and compared the empirical perturbation results to the predicted ones.

Existing perturbation inference algorithms can be divided into two major classes: (i) algorithms that assume binary states for molecular participants in the pathway (e.g. affected vs. not affected) (22) and (ii) algorithms that apply multiple discrete states to the participants (e.g. upregulated, downregulated and no change) (23). One of the most widely used pathway inference tools in cancer genomics that relies on binary states is HotNet (24), which uses a thermal diffusion model to identify regions of influence of affected and unaffected genes. While effective for identifying mutations in different patients with similar pathway-level effects, HotNet has difficulty predicting the combined effect of co-occurring mutations or producing testable predictions on downstream pathway activity. The most popular pathway inference tool that relies on multiple discrete states is PARADIGM (25). PARADIGM allows the users to supply the state (downregulated, unchanged, and upregulated) of known genes, RNAs or proteins to protein-protein interaction (PPI) networks. The core mathematical algorithm used by PARADIGM is the loopy-belief-propagation (26), which is provided by the C++ library libDAI (https://github.com/dbtsai/libDAI) (27). PARADIGM is capable of inferring the output nodes which have been affected by upstream perturbations. It takes into consideration the combined effect from multiple perturbed sources on a single output by increasing the confidence of the output being perturbed when multiple converging input nodes are perturbed. By relying on the PPI networks, however, PARADIGM does not consider the elaborate biochemical relationships between PPI participants, nor does it provide a direct way to evaluate perturbation intensity.

MP-BioPath applies a non-linear optimization model to a directed chain of reactions in a pathway, from root pathway inputs to terminal pathway outputs, through intermediary products. Thus, MP-BioPath algorithm represents an improvement over both a two-state or discrete state algorithm and provides a setup for the application of dynamic biochemical parameters.

We found a high degree of concordance between the empirical and predicted perturbation effects. The concordance ranged from 56% (curator)/44% (MP-BioPath) to 100% (curator) /94% (MP-BioPath), with a mean accuracy of 81% (curator)/75% (MP-BioPath). There was no statistically significant difference in accuracy, sensitivity, and specificity between curator-based and MP-BioPath-based predictions (Table 3 and Supplementary Table S6). When we examined discrepant curator-based predictions, we found that the great majority (95%) of discrepant predictions were due to gaps in the knowledgebase, i.e. molecules and relationships that had not yet been curated, and only 5% of discordant cases (7 tests total) could be attributed to incorrect annotations in the knowledgebase (Figure 5). Our examination of discrepant predictions by MP-BioPath reached similar conclusions, except that a modest number of additional discrepancies were generated when the algorithm predicted a change in the correct direction which fell below the threshold we used for discretizing its continuous output activity values. This latter issue was exacerbated among pathways that contained ‘entity sets’ which represent multiple molecules or complexes that play equivalent roles in a reaction, such as tissue-specific isozymes. In such cases, MP-BioPath applies the same effect weights to all members of the set, thereby diluting the effect of any individual member and diminishing the output effect value. This issue will be addressed in future iterations of the algorithm by allowing the weights of set members to be adjusted based on their relative expression in the tissue of interest.

Besides the knowledge gaps that are intrinsic to any biological database and the disadvantage of comparing empirical data, which is usually generated in a small number of cell types, with in silico simulations in a generic cell, it should be noted that the methods used in this study have additional limitations. First, literature search itself is limited by the inconsistent use of gene and protein names, dependency on author-specified keywords, default settings of the search engine, and researcher bias, which increases the propensity for human-introduced errors when creating the ground truth for comparison. A more fundamental challenge arises from the arbitrary boundaries of biological pathways (28, 29). In the context of a pathway database such as Reactome, a biological process is often not shown within a single pathway diagram but is spread over several sub-pathway diagrams connected via flow links. For example, the ‘RAF/MAP kinase cascade’ is composed of many upstream feeder pathways. These feeder pathways usually show the formation of the active RAS:GTP complex, and then direct users to the ‘RAF/MAP kinase cascade’ diagram. Experimentally, however, activation of RAS signaling is usually detected not by measuring the amount of GTP-bound RAS, but by checking the phosphorylation status of downstream MAP kinases MAPK1 (ERK2) and MAPK3 (ERK1). For this reason, some of the false negatives we encountered may have arisen from test cases in which the biological path crossed sub-pathway boundaries and a key connection was omitted from the logic diagram. This problem could be mitigated with a more robust algorithm to generate logic diagrams that include downstream events defined in the Reactome knowledgebase but not visually displayed in a single diagram, potentially coupled with curation to improve the alignment of pathway boundaries with the standard experimental readouts used to test the pathway’s activity.

For both curator and MP-BioPath predictions, we observed a significant increase in accuracy for the prediction of posttranslational modification events relative to prediction of GER events. We interpret this observation as reflecting Reactome’s better coverage of posttranslational modifications than transcriptional regulation. One factor explaining this is that GER is dependent on dosage of transcription factors and other quantitative parameters, such as the affinity of transcription factors for their different target sites (30), that Reactome currently does not capture. Another factor is that the various modes of regulation of a particular gene may be assigned to different pathways due to the assignment of arbitrary pathway boundaries described earlier.

In addition, the fact that some of the literature references providing evidence of perturbation effects had been cited by Reactome during pathway curation did positively affect the accuracy of both curator and MP-BioPath predictions. Namely, 298/847 cases were supported by at least one of the 158 publications cited by Reactome. Although the fraction of published perturbation effect studies cited by Reactome does not significantly correlate with the predictive accuracy of individual pathways (Supplementary Figure S6E), when looking at these 298 test cases in isolation, their predictive accuracy calculated from the Supplementary Table S1, was 95% for curators and 91% for MP-BioPath. The future use of high-throughput perturbation datasets as experimental readouts will provide a more detailed evaluation of Reactome pathway-based predictive accuracy, removing some of the bias introduced by literature search.

In summary, these results add to our confidence in the utility of pathway knowledgebases for predicting the effects of pathway perturbations and should be encouraging to ongoing efforts to create effective fully automated pathway-based predictive models for use in drug target discovery, mutation effect prediction, and synthetic lethality discovery (25, 31, 32). In addition, the study shows that pathway databases such as Reactome, by grouping biochemical reactions into causal chains (pathways), reveal distant relationships between genes, proteins, and small molecules that may not have been experimentally explored, which has the potential to fuel hypothesis-driven research.

## Supplementary Material

baac009_Supp

## Data Availability

The tests have been compiled and made publicly available in the following GitHub repository: https://github.com/OICR/mp-biopath-reactome-ten-pathway-tests/releases/tag/1.0.0. The Logical Networks themselves can be found within the ‘pathways’ folder in this repository. Supplementary tables can be downloaded as an xlsx file from the GitHub repository (each spreadsheet in the file represents one supplementary table): https://github.com/OICR/mp-biopath-reactome-ten-pathway-tests/blob/main/PredictiveAccuracyOfBiologicalPathways_SupplementaryTables.xlsx. The script used for generating the Logical Networks from Reactome V66 Pathways can be found in GitHub here: https://github.com/reactome/Release/blob/master/scripts/reaction_logic_table.pl. The version of MP-BioPath that was used to perform the analysis is 1.0.4 and can be found in the following GitHub repository: https://github.com/OICR/mp-biopath/releases/tag/1.0.4. This version of MP-BioPath can also be accessed through pre-provisioned Docker containers in the following DockerHub repository: https://hub.docker.com/r/oicr/mpbiopath.
