# Supplementary material for: Evaluating the predictive accuracy of curated biological pathways in a public knowledgebase
Source: Database (Oxford). 2022 Mar 6;2022:baac009. doi: 10.1093/database/baac009 (PMC9216552; doi:10.1093/database/baac009)
Supplement: baac009_Supp [file baac009_supp.zip › PredictiveAccuracyOfBiologicalPathways_SupplementaryFigures.pptx]

## Slide 1
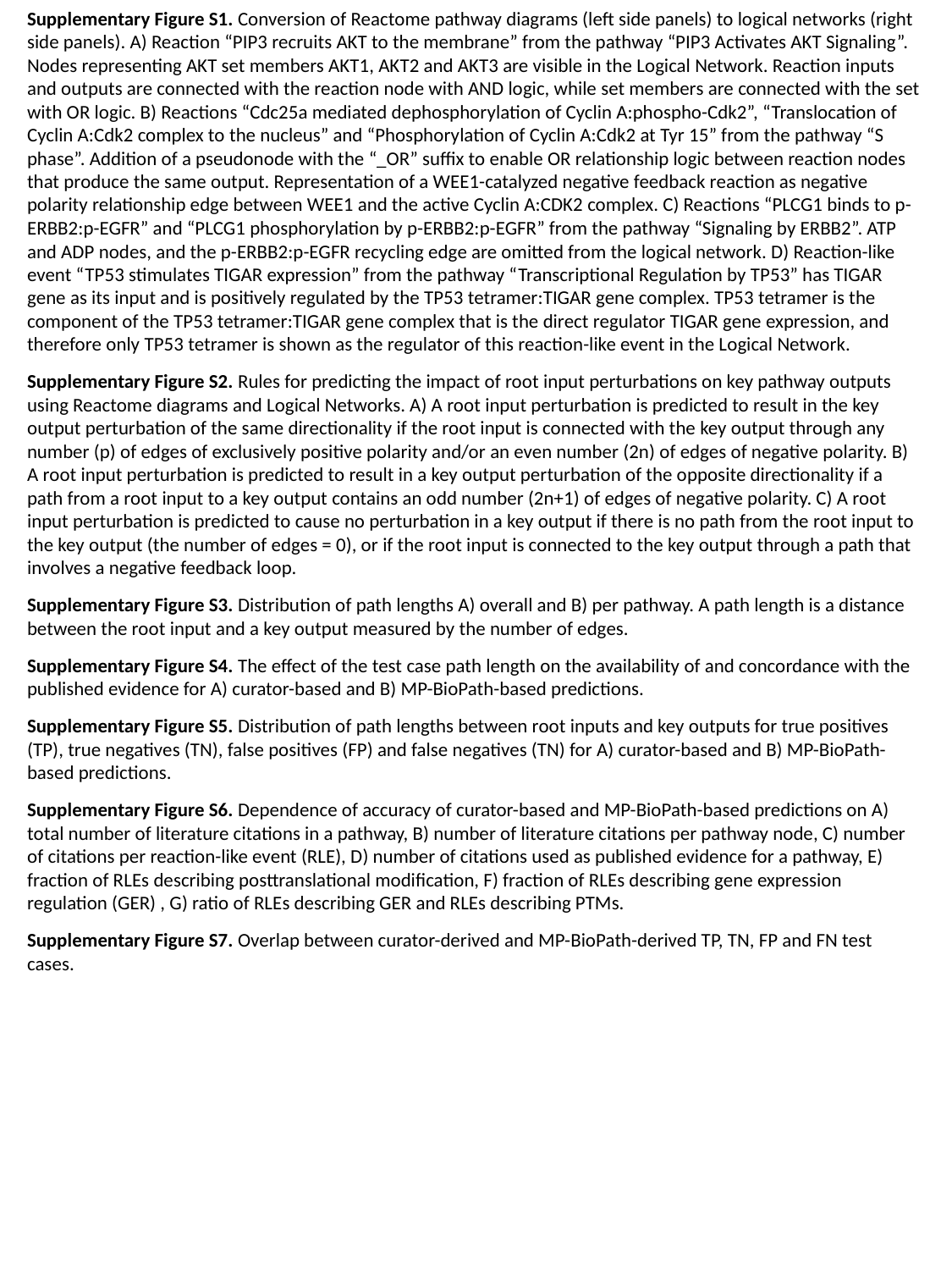

Supplementary Figure S1. Conversion of Reactome pathway diagrams (left side panels) to logical networks (right side panels). A) Reaction “PIP3 recruits AKT to the membrane” from the pathway “PIP3 Activates AKT Signaling”. Nodes representing AKT set members AKT1, AKT2 and AKT3 are visible in the Logical Network. Reaction inputs and outputs are connected with the reaction node with AND logic, while set members are connected with the set with OR logic. B) Reactions “Cdc25a mediated dephosphorylation of Cyclin A:phospho-Cdk2”, “Translocation of Cyclin A:Cdk2 complex to the nucleus” and “Phosphorylation of Cyclin A:Cdk2 at Tyr 15” from the pathway “S phase”. Addition of a pseudonode with the “_OR” suffix to enable OR relationship logic between reaction nodes that produce the same output. Representation of a WEE1-catalyzed negative feedback reaction as negative polarity relationship edge between WEE1 and the active Cyclin A:CDK2 complex. C) Reactions “PLCG1 binds to p-ERBB2:p-EGFR” and “PLCG1 phosphorylation by p-ERBB2:p-EGFR” from the pathway “Signaling by ERBB2”. ATP and ADP nodes, and the p-ERBB2:p-EGFR recycling edge are omitted from the logical network. D) Reaction-like event “TP53 stimulates TIGAR expression” from the pathway “Transcriptional Regulation by TP53” has TIGAR gene as its input and is positively regulated by the TP53 tetramer:TIGAR gene complex. TP53 tetramer is the component of the TP53 tetramer:TIGAR gene complex that is the direct regulator TIGAR gene expression, and therefore only TP53 tetramer is shown as the regulator of this reaction-like event in the Logical Network.
Supplementary Figure S2. Rules for predicting the impact of root input perturbations on key pathway outputs using Reactome diagrams and Logical Networks. A) A root input perturbation is predicted to result in the key output perturbation of the same directionality if the root input is connected with the key output through any number (p) of edges of exclusively positive polarity and/or an even number (2n) of edges of negative polarity. B) A root input perturbation is predicted to result in a key output perturbation of the opposite directionality if a path from a root input to a key output contains an odd number (2n+1) of edges of negative polarity. C) A root input perturbation is predicted to cause no perturbation in a key output if there is no path from the root input to the key output (the number of edges = 0), or if the root input is connected to the key output through a path that involves a negative feedback loop.
Supplementary Figure S3. Distribution of path lengths A) overall and B) per pathway. A path length is a distance between the root input and a key output measured by the number of edges.
Supplementary Figure S4. The effect of the test case path length on the availability of and concordance with the published evidence for A) curator-based and B) MP-BioPath-based predictions.
Supplementary Figure S5. Distribution of path lengths between root inputs and key outputs for true positives (TP), true negatives (TN), false positives (FP) and false negatives (TN) for A) curator-based and B) MP-BioPath-based predictions.
Supplementary Figure S6. Dependence of accuracy of curator-based and MP-BioPath-based predictions on A) total number of literature citations in a pathway, B) number of literature citations per pathway node, C) number of citations per reaction-like event (RLE), D) number of citations used as published evidence for a pathway, E) fraction of RLEs describing posttranslational modification, F) fraction of RLEs describing gene expression regulation (GER) , G) ratio of RLEs describing GER and RLEs describing PTMs.
Supplementary Figure S7. Overlap between curator-derived and MP-BioPath-derived TP, TN, FP and FN test cases.

## Slide 2
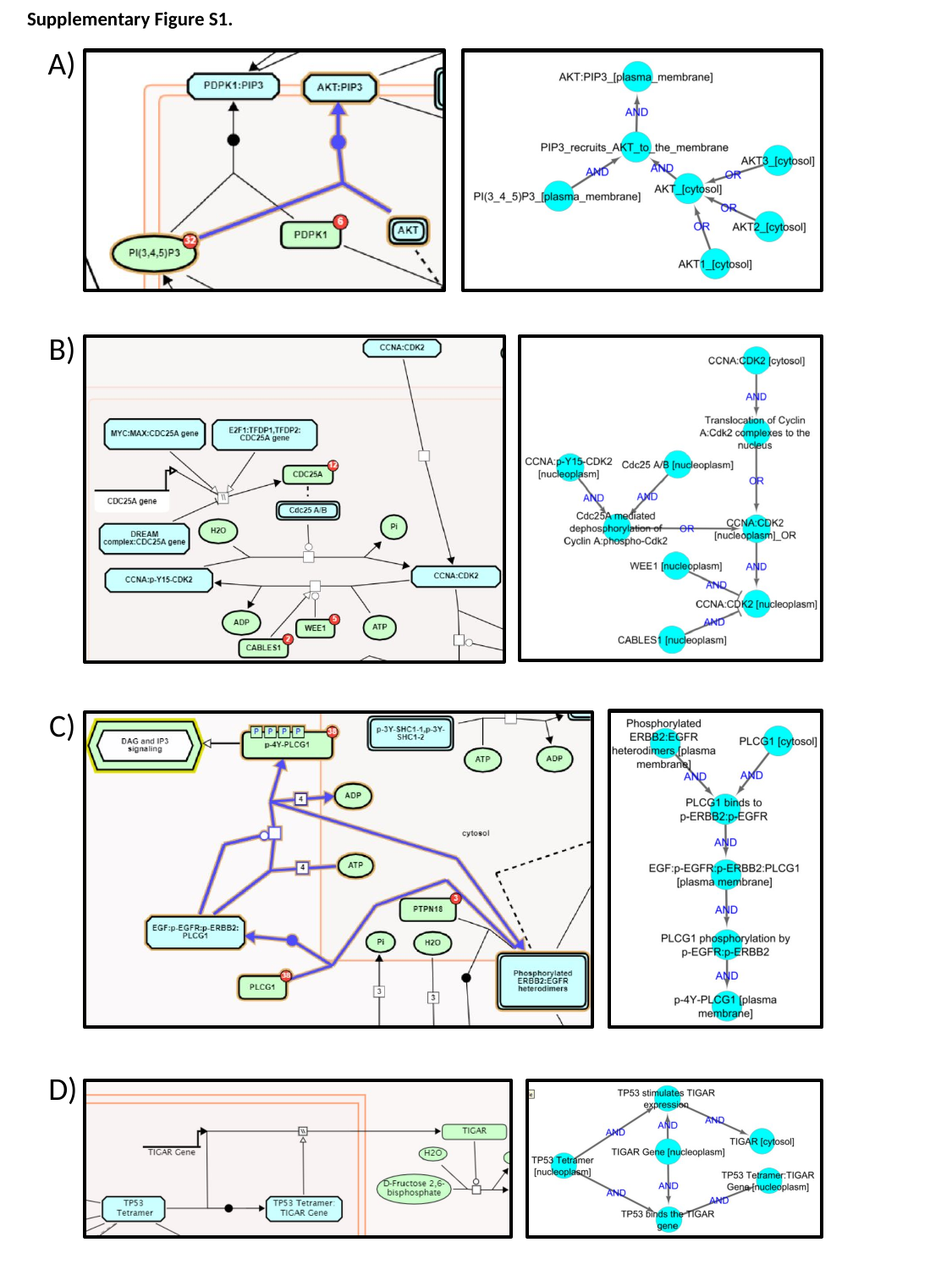

Supplementary Figure S1.
A)
B)
C)
D)

## Slide 3
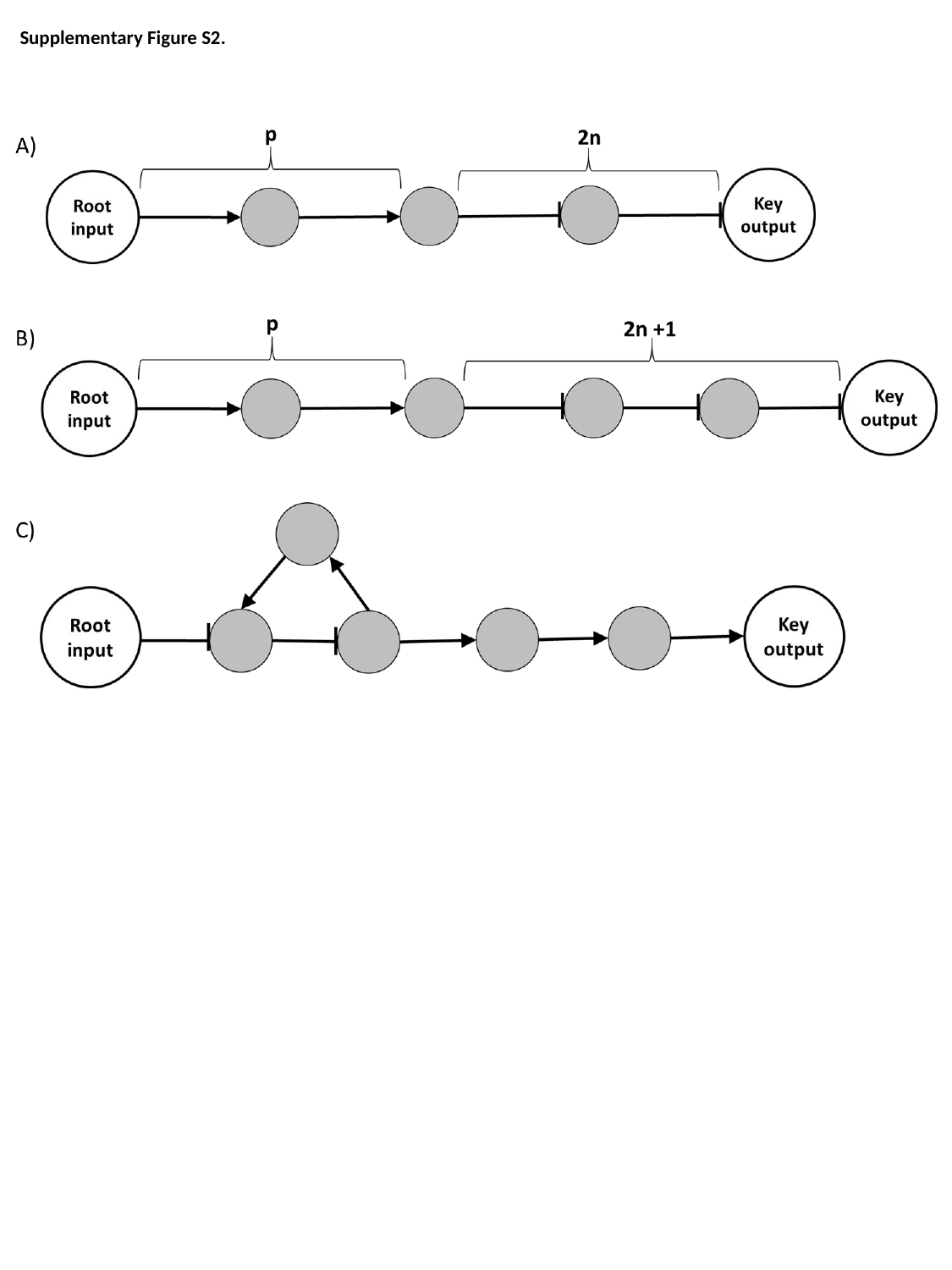

Supplementary Figure S2.

## Slide 4
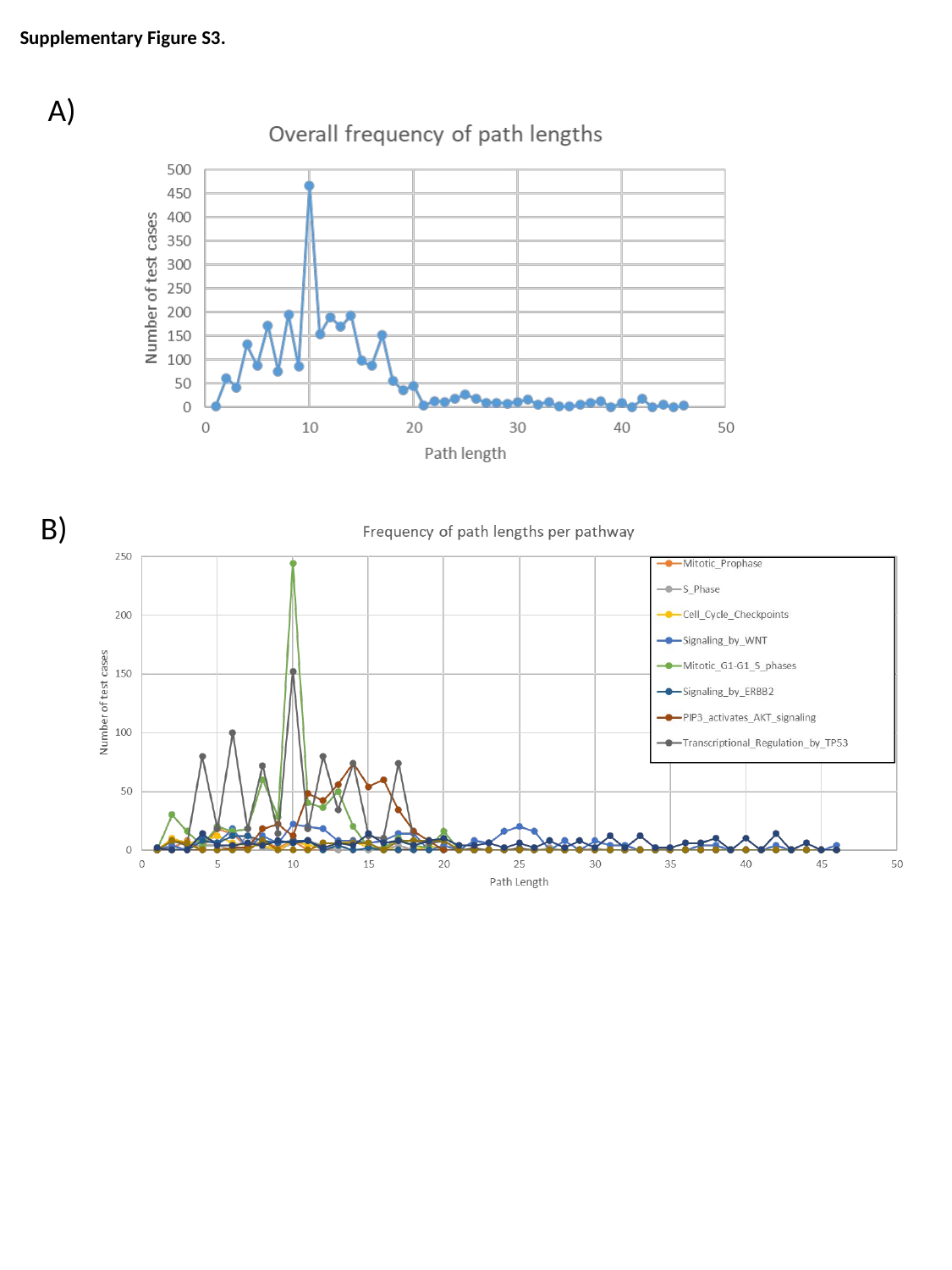

Supplementary Figure S3.
A)
B)

## Slide 5
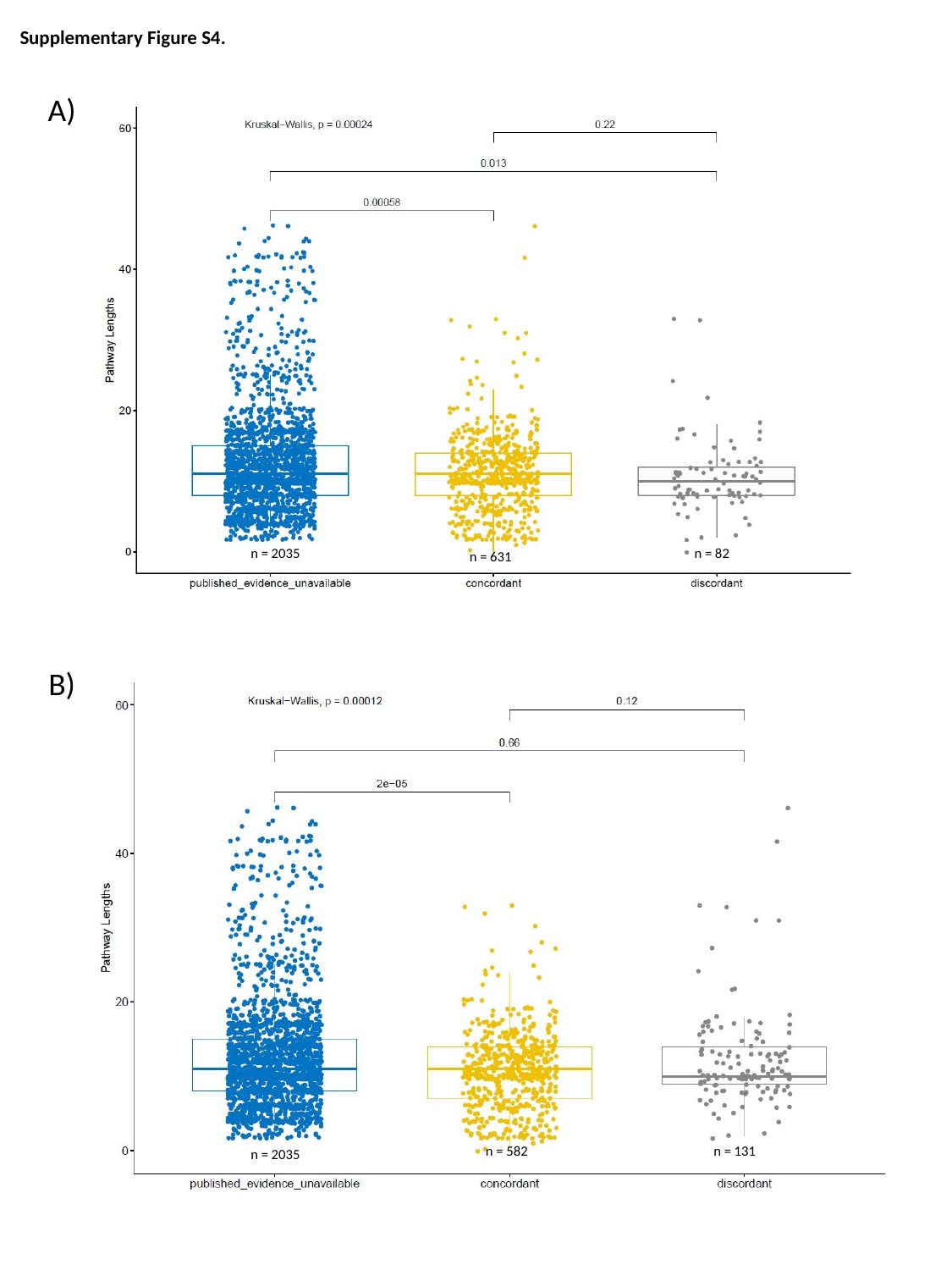

Supplementary Figure S4.
A)
n = 2035
n = 82
n = 631
B)
n = 582
n = 131
n = 2035

## Slide 6
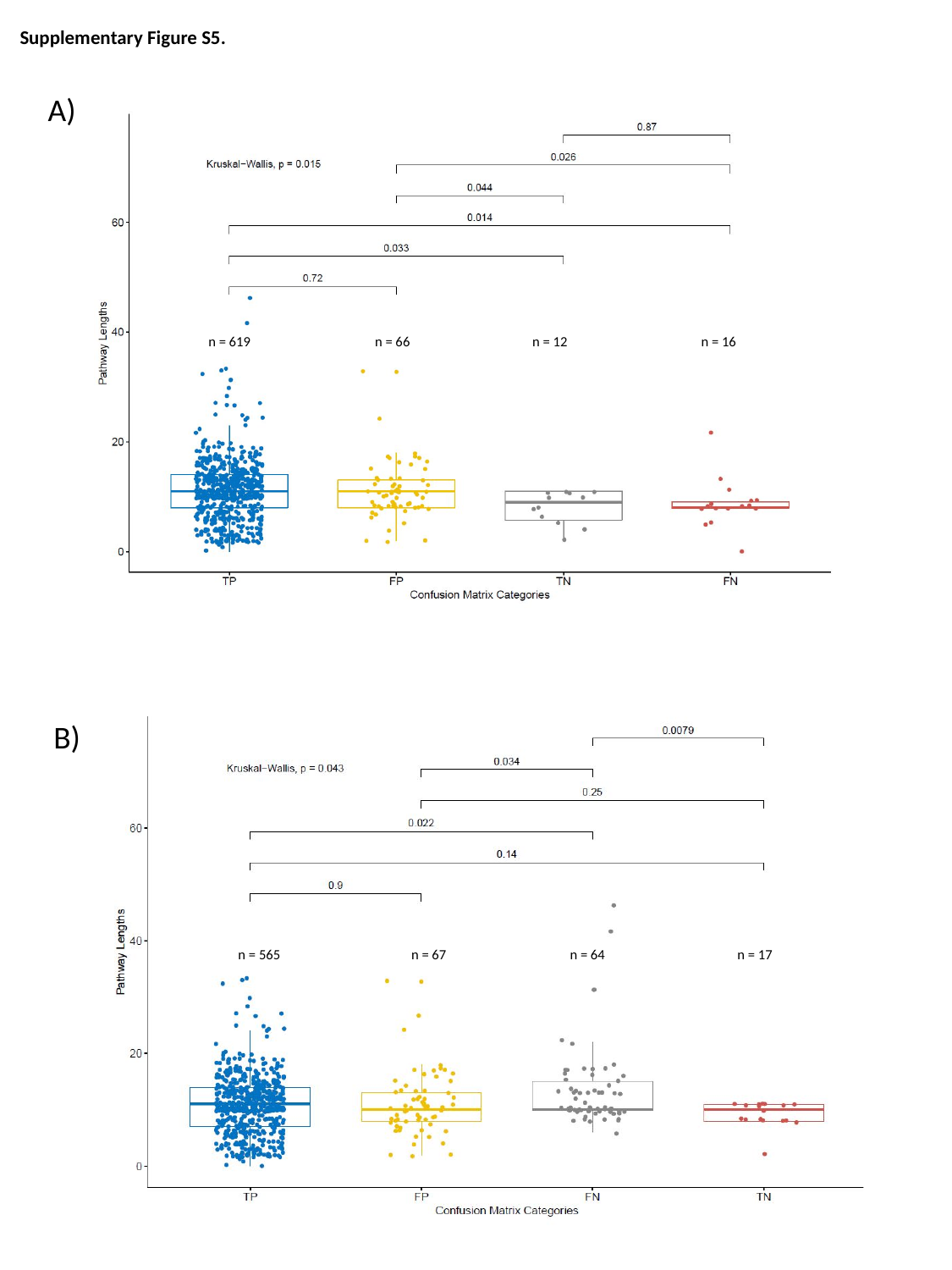

Supplementary Figure S5.
A)
n = 619
n = 66
n = 12
n = 16
B)
n = 565
n = 67
n = 64
n = 17

## Slide 7
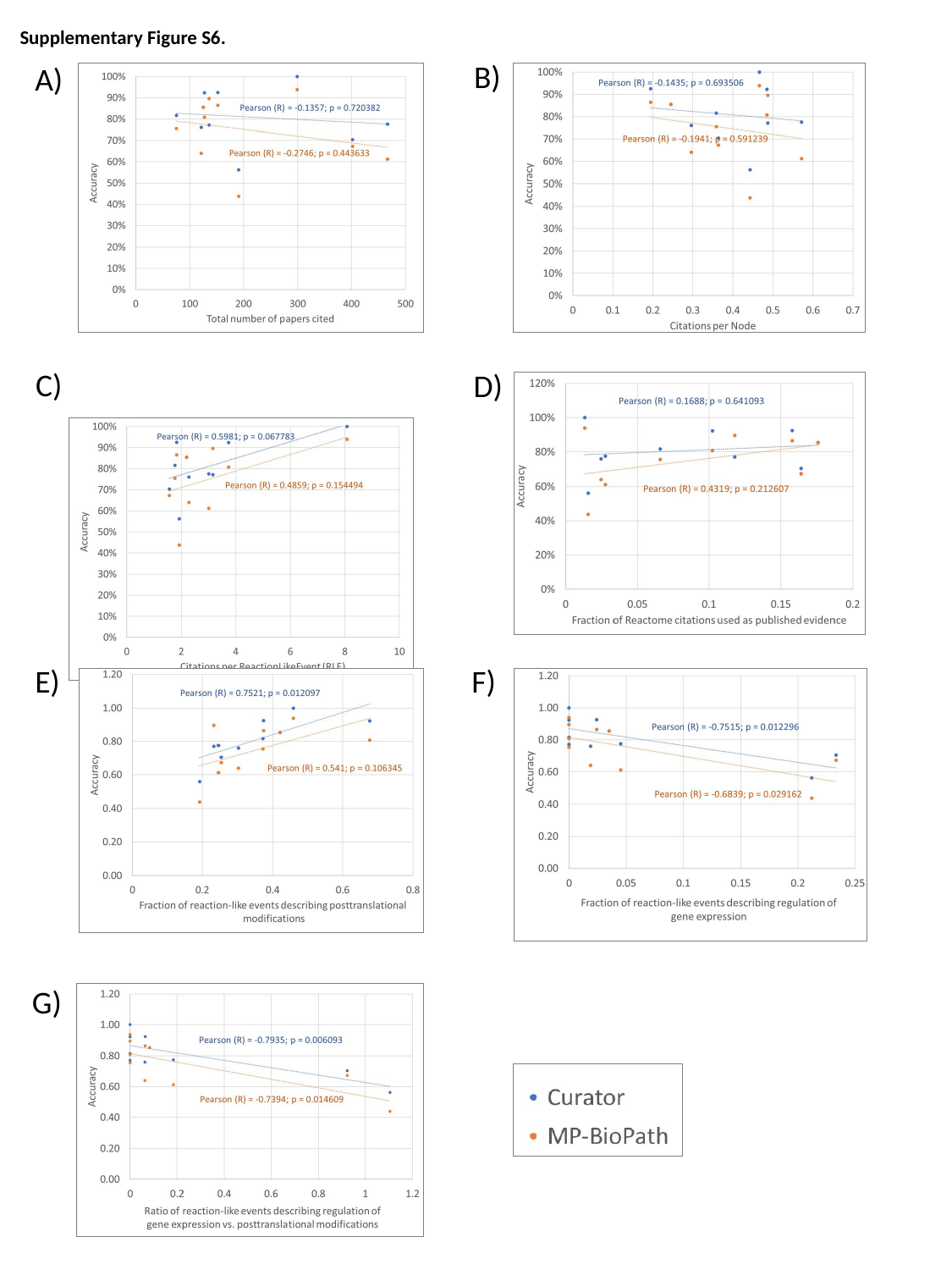

Supplementary Figure S6.
B)
A)
C)
D)
E)
F)
G)

## Slide 8
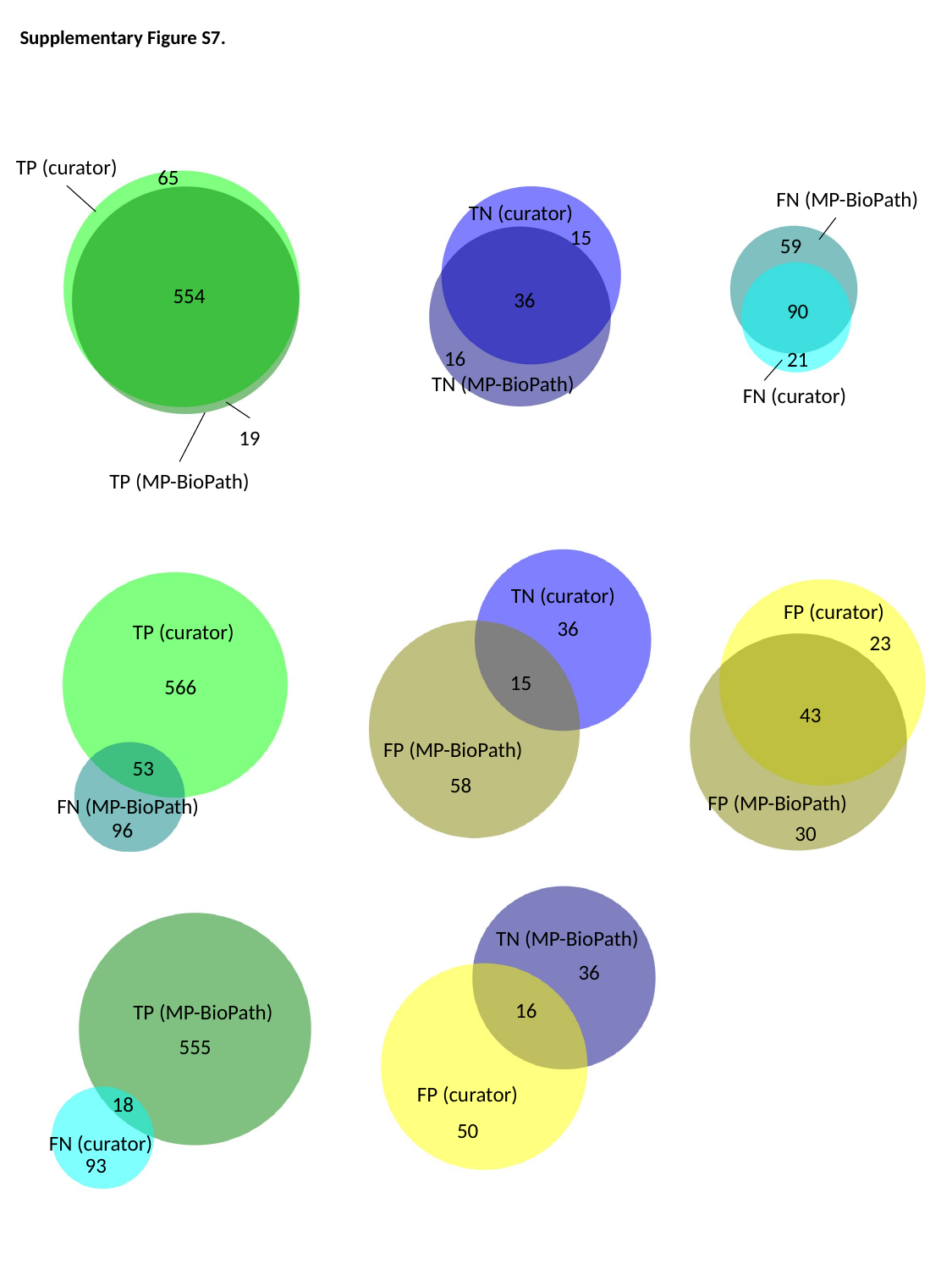

Supplementary Figure S7.
TP (curator)
65
554
19
TP (MP-BioPath)
TN (curator)
15
36
16
TN (MP-BioPath)
FN (MP-BioPath)
59
90
21
FN (curator)
TN (curator)
36
15
FP (MP-BioPath)
58
TP (curator)
566
53
FN (MP-BioPath)
96
FP (curator)
23
43
FP (MP-BioPath)
30
TN (MP-BioPath)
36
16
FP (curator)
50
TP (MP-BioPath)
555
18
FN (curator)
93
