# Supplementary material for: Evaluating the predictive accuracy of curated biological pathways in a public knowledgebase
Source: Database (Oxford). 2022 Mar 6;2022:baac009. doi: 10.1093/database/baac009 (PMC9216552; doi:10.1093/database/baac009)
Supplement: baac009_Supp [file baac009_supp.zip › PredictiveAccuracyOfBiologicalPathways_SupplementaryMethods.docx]

# Supplementary Methods

## Converting Reactome Pathways to Logical Networks

Logical Networks consist of input (I) and output (O) node pairs and their connecting edges, characterized by fundamental edge attributes. Nodes in these networks represent either physical entities (e.g. individual gene products or macromolecular complexes) or reactions, while edges represent either the physical relationships among individual molecules in a complex or a set, or the functional relationships among the inputs, outputs, catalysts and regulators participating in a reaction. Nodes representing complexes and sets are broken down into their constituent parts resulting in a more granular representation of the pathway (Supplementary Figure 1A).

The two fundamental relationship attributes in the Logical Networks are the polarity and the relationship type. A positive polarity implies that an increase in activity of the upstream node increases the activity of the downstream node and a negative polarity implies a negative interaction. For example, an edge connecting an enzyme with a reaction it catalyzes is assigned a positive polarity, while an edge between an inhibitor and a reaction it inhibits is assigned a negative polarity. The relationship type is either an AND or an OR, which indicates how the effects of multiple edges entering a node should be logically combined. For example, all the components of a protein complex have an AND relationship with the complex because each is normally required for the complex to form. Some physical entities are functionally interchangeable with each other in a particular pathway context, in which case they have an OR relationship when entering a complex or reaction. In particular, these OR relationships occur with input set members and their output set (Supplementary Figure 1A) and with input reaction nodes and their outputs in the event that two or more reactions in a pathway generate the same product (Supplementary Figure 1B).

Reactome pathway diagrams frequently feature physiological positive and negative feedback loops, some of which can only be identified reliably by reading curator-generated descriptions of reactions and pathways. In order to mark negative feedback loops in Logical Networks, the automated Logical Networks were simplified by manually creating a negative relationship edge connecting the catalysts and/or positive regulators of negative feedback loops with the input entities they downregulate (Supplementary Figure 1B).

A feature of Reactome pathways is recycling loops, which occur when, for example, a protein participates in a binding reaction to form a complex and subsequently dissociates from that complex. As Reactome does not contain quantitative information, it is not possible to know if a recycling loop is rate-limiting. While recycling loops cannot be visually distinguished from positive feedback loops in Reactome diagrams and Logical Networks, they can be computationally identified via curator-defined preceding-following event relationships in the Reactome database. During Logical Networks construction, recycling loops are resolved automatically by removing an edge connecting a reaction node R_n_ to its output O_Rn_ if O_Rn_ represents a recycled physical entity that was used as an input I_Rn-i_ (therefore O_Rn_ = I_Rn-i_) for reaction R_n-i_ that serves as a predecessor reaction for R_n_ in a chain of n reactions, unless R_n_ has been manually annotated as a preceding event of R_n-i_ (that is, unless R_n-i_ and R_n_ are part of a positive feedback loop). Automated resolution of recycling loops also involves the omission of frequent simple molecule inputs and outputs from the Logical Networks (e.g. water, ATP, ADP) (Supplementary Figure 1C). When converting Reactome pathways to Logical Networks, additional recycling loops not present in Reactome pathway diagrams may be generated by breaking down root input complexes and sets into their constituents. A recycled constituent is represented by manually duplicating it and appending the suffix "_c" to its name in the Logical Networks. In cases where a reaction input is simultaneously a component of a complex that acts as a positive or negative regulator of the reaction, because the formation of a complex between a direct regulator and the reaction input is a prerequisite for the regulation, only the designated direct regulator was exported to the Logical Networks to avoid the creation of artifactual positive feedback loops (Supplementary Figure 1D).

In Logical Networks, root input (RI) nodes and terminal output (TO) nodes were identified in a semi-automated fashion by applying BioVenn [(33)](https://paperpile.com/c/ZSo3AT/M2zeq) to determine the intersection (I ∩ O) of all Logical Networks input (I) and output (O) nodes.

The number of edges between root inputs and key outputs (path length) was determined by PathLinker [(12)](https://paperpile.com/c/ZSo3AT/vcxUk). When several directed paths existed between a root input and a key output, only the shortest path length was included in Supplementary Table S1.

## MP-BioPath Description

MP-Biopath is a command-line tool that implements the mathematical optimization model described in the Materials and Methods section2.6 and is written in the Julia programming language. Julia is a high performance dynamic open-source programming language designed for computation [(34)](https://paperpile.com/c/ZSo3AT/qubWC). One of the main groups developing modules for the Julia community are those involved in Operations Research [(35)](https://paperpile.com/c/ZSo3AT/ruS7L). One particular module that facilitates the development of optimization models and works with many major solvers is the JuMP library. This library allows a developer to implement the model programmatically in Julia and then run the model on one of the many possible solvers [(34)](https://paperpile.com/c/ZSo3AT/qubWC).

The solver used in this implementation is from the Computational Infrastructure for Operations Research (COIN-OR). This initiative provides several open-source operations research applications [(36)](https://paperpile.com/c/ZSo3AT/imHLh). From this initiative, several solvers were tested. The interior-point solver IPOpt is used by MP-BioPath and is capable of handling all the features present in the mathematical model, including the multiplication of decision variables together within the constraint and squaring expressions in the minimization formula [(37)](https://paperpile.com/c/ZSo3AT/Zy46l). IPOpt can also be configured to be run on Graphics Processing Units (GPUs) for higher performance.

The tool can be run inside or outside of the provided Docker image [(38)](https://paperpile.com/c/ZSo3AT/twOqJ). Before running MP-BioPath, the user needs to configure each run through configuration files, as described in the documentation (https://oicr.gitbooks.io/mp-biopath-documentation/content/). Once the user has created the configuration file, they can run MP-BioPath with the following command “*julia bin/mp-biopath inference --config <your-config.yaml>”*.
